# Embedding textile capacitive sensing into smart wearables as a versatile solution for human motion capturing

**DOI:** 10.1038/s41598-024-66165-z

**Published:** 2024-07-09

**Authors:** Daniel Geißler, Bo Zhou, Hymalai Bello, Joanna Sorysz, Lala Ray, Hamraz Javaheri, Matthias Rüb, Jan Herbst, Esther Zahn, Emil Woop, Sizhen Bian, Hans D. Schotten, Gesche Joost, Paul Lukowicz

**Affiliations:** 1 Embedded Intelligence, German Research Center for Artificial Intelligence (DFKI), Kaiserslautern, Germany; 2 Intelligent Networks, German Research Center for Artificial Intelligence (DFKI), Kaiserslautern, Germany; 3 Design Research eXplorations, German Research Center for Artificial Intelligence (DFKI), Berlin, Germany; 4 Information Technology and Electrical Engineering, ETH Zurich, Zurich, Switzerland

**Keywords:** Electrical and electronic engineering, Sensors and biosensors

## Abstract

This work presents a novel and versatile approach to employ textile capacitive sensing as an effective solution for capturing human body movement through fashionable and everyday-life garments. Conductive textile patches are utilized for sensing the movement, working without the need for strain or direct body contact, wherefore the patches can sense only from their deformation within the garment. This principle allows the sensing area to be decoupled from the wearer’s body for improved wearing comfort and more pleasant integration. We demonstrate our technology based on multiple prototypes which have been developed by an interdisciplinary team of electrical engineers, computer scientists, digital artists, and smart fashion designers through several iterations to seamlessly incorporate the technology of capacitive sensing with corresponding design considerations into textile materials. The resulting accumulation of textile capacitive sensing wearables showcases the versatile application possibilities of our technology from single-joint angle measurements towards multi-joint body part tracking.

## Introduction

Human motion capturing is an essential research field with many established tracking possibilities, mainly from the computer vision and wearable community, to reliably track individual human body movements. Each of the existing methodologies typically accompanies several disadvantages, making it difficult to identify one of them as the most suitable one. Computer Vision solutions, marker or marker-less-based, usually lack flexibility in terms of human ground movement like walking or running, due to the necessity of calibrated and stable camera setups^1,2^. The wearable tracking technology, for instance, strain-based sensors or Inertial Measurement Units (IMU), require fixed positioning on the user’s skin, resulting in complex calibrations and wearing discomfort^3,4^. Additionally, the current wearable market focuses on rigid accessories like smartwatches and glasses, while research has a growing interest in textile-integrated functions^5,6^.

In contrast, Capacitive Sensing offers the unique advantage of embedding the sensing principle in a natural and comfortable way directly into the garments without fixed positioning on the skin^7^. The possibility and versatility for capacitive sensing becomes apparent in this work, especially in the area of applications where present technologies e.g. visual systems reach their limits when calibration or the utilization of external tracking systems is not applicable. Especially in niches such as medical rehabilitation or professional athlete training usually requiring^8^.

Its key functions of wearables to enable interactions and life logging are sensing the correlation of textile movements and the user’s activities^9^. The centerpiece of our technology consists of conductive textile patches, which can be integrated into the garments individually. Based on the sensing patches’ shape and position, the capacitive change, caused by contact, proximity, or the garment’s deformation itself while moving can be measured, resulting in a strain-less and skin-contact-free tracking technology with minimum discomfort. The same patch can be used for multiple purposes with the help of machine learning to recognize temporal pattern characteristics of different interaction and activity contexts.

The main contributions of this work can be summarized as follows:

- We illustrate that Textile Capacitive Sensing is a valid alternative to existing human motion tracking systems by outlining the technical capabilities, potential, and accuracy.
- We present the diverse concepts of Textile Capacitive Sensing through multiple prototypes, starting from joint angle measurements over body segment tracking towards full body capturing.
- We additionally focus on the aggregation of technical smart wearables and their design aspects to make the garments more appealing than simple proof-of-concept prototypes.

## Wearable textile capacitive sensing technology

### Background

Many studies have investigated using specialized materials to detect human motions, such as piezoelectric materials^10^, MXene/Ag nanowires^11,12^, photodetector materials^8^, triboelectric materials^13^ and others. However, those methods rely on highly specialized and costly materials with special properties, and careful placement of different materials to satisfy the sensing principles. These requirements limit the broad dissemination and democratization of smart textile wearables. Each material also requires unique electronic drivers, making them not easily interchangeable.

Capacitive sensing, a widely used physical-to-digital conversion technique in electronics, on the other hand, requires only materials with conductivity similar to the metals that form regular circuits. This requirement can be sufficed not only by specially nano-coated materials^14^, but also off-the-shelf conductive yarns, and EM shielding fabrics available to the public. This makes capacitive sensing a viable option for a broad portfolio of applications. There are generally two types of capacitance measurement solutions depending on the principle: current-based and frequency-based. As capacitance variation is essentially a process of charge redistribution on the conductive plates, by observing the charge flow (current), the capacitance variation could be deduced. A high input impedance with a high amplification circuit is usually used to sense the charge flow as the current flow is very subtle^15^. Compared with frequency-based capacitive sensing, the charge-based solution consumes much less power but is prone to EM interference.

Therefore, frequency-based capacitance measurement is widely used. As a capacitor is an integral component of many oscillators, there is a definitive relationship between the resonant frequency/amplitude and capacitance in an oscillator. By introducing the surroundings into an oscillator through an antenna, the motion-related capacitance value can be derived by either reading the envelope of the oscillating AC signal after modulation^16^ or counting the frequency of the oscillating AC signal directly^17,18^. For example, in^16^, the author designed an envelope detector composed of a diode and low pass filter followed by a Colpitts Oscillator, and the measured amplitude information is used for capacitance perceiving.

### Textile integration and usages

**Figure 1:**
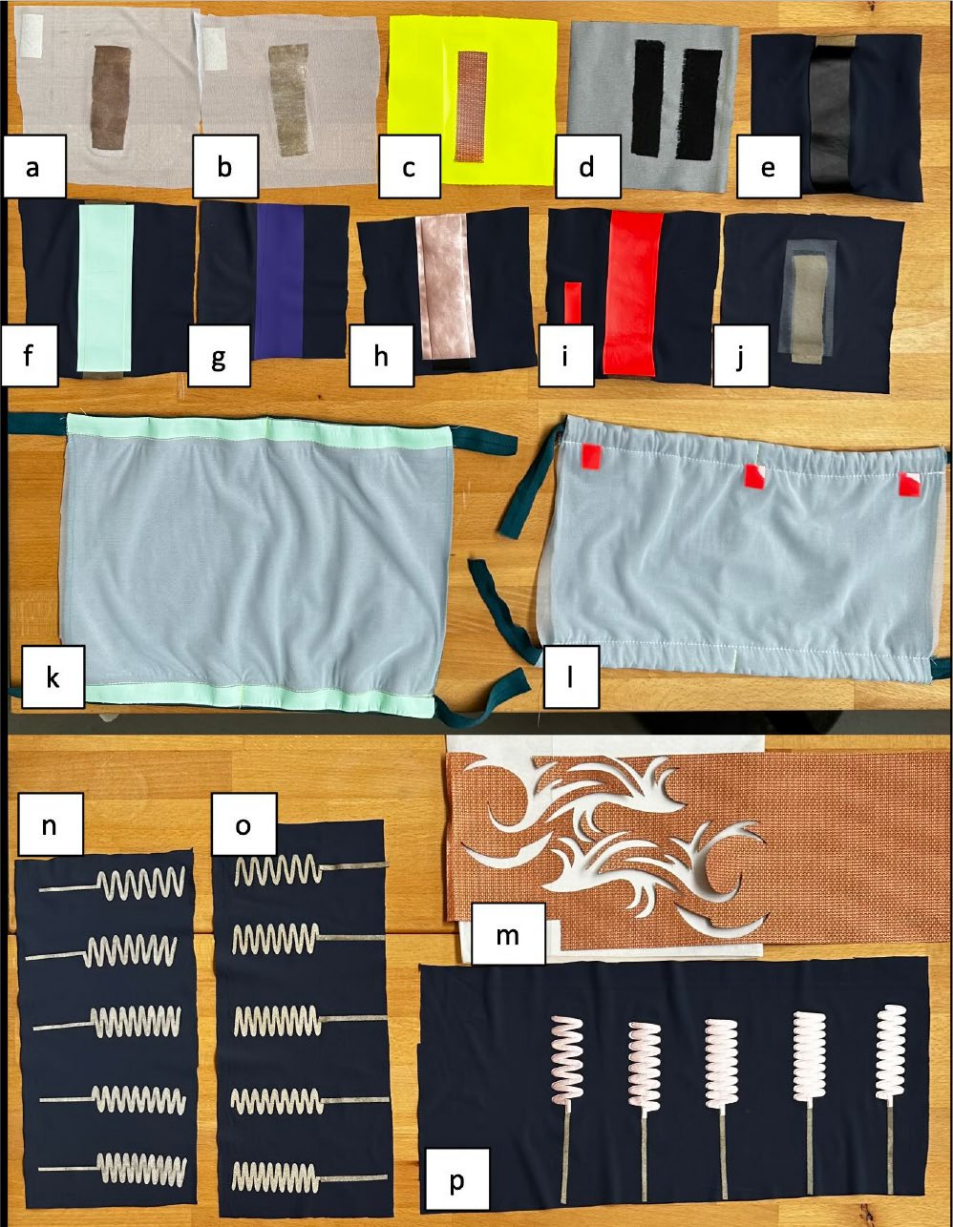
Testing different combinations of substrate, sensing, and cover materials using heat transfer technology.

**Figure 2:**
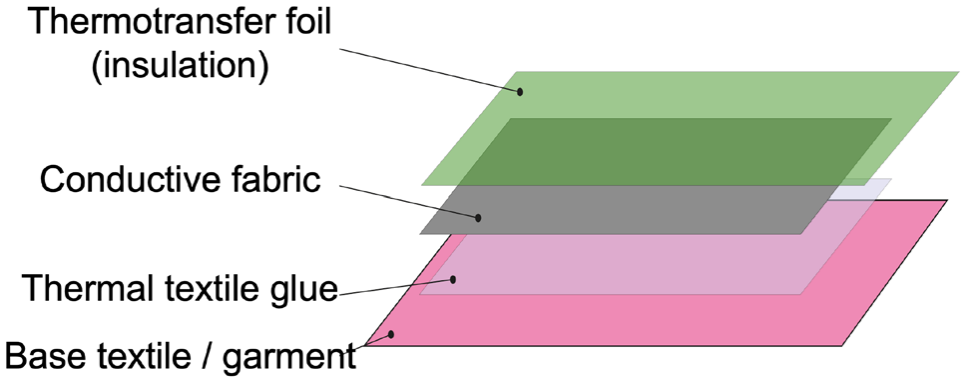
The layer stack of the protected textile capacitive sensor patch.

**Figure 3:**
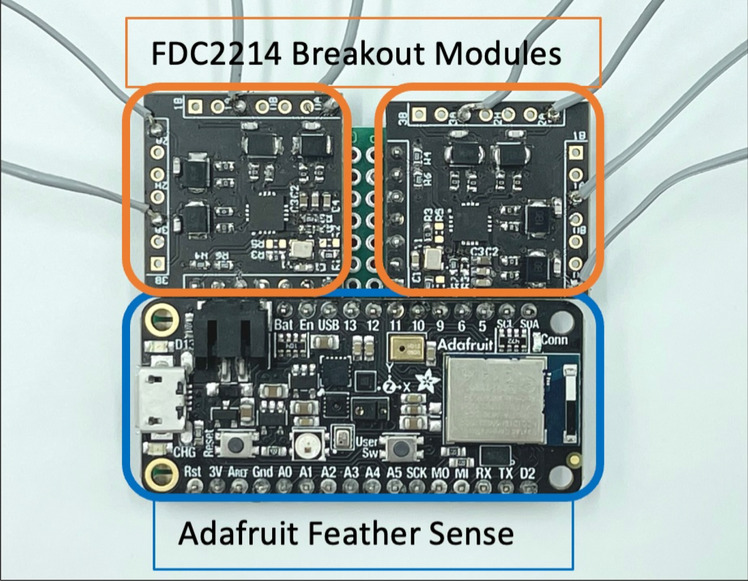
Breakout module of the Data Acquisition Unit^19^.

The basis for including the proposed capacitive sensing functionality in a garment rests on the conductive fabrics and their characteristics. Such material can be used as the antenna to form the capacitor, which can further be applied in downstream tasks such as motion capturing or gesture recognition similar to a Theremin^20^. To connect the conductive patches with the data acquisition unit (DAU) for sensing the capacitance, conductive traces from the same material can be used to route through the garment^19^. Conductive traces have proven to be more robust than using single core wires for each patch due to the extensive movement in the clothing and the accompanying fatigue of the thin wires. A well-established method to apply the chosen material to textiles is heat transferring it directly onto the garment. By stacking up the textile substrate, the conductive material, a thermotransfer foil as isolating cover to add robustness, and adding some thermal textile glue, the heat pressing fully bonds the materials into one cohesive textile as shown in Fig. 2. There are basically two setups of patches and traces that form the sensing pipeline together with the DAU. The traces themself can be used as sensing fabric to cover bigger areas equally by routing through the garment. Using bigger patches and thinner traces as connecting parts on the contrary places shifts the sensing focus more on the explicitly chosen body part for tracking.

The DAU is shown in Fig. 3 as a breakout module for visualization. During our development through multiple iterations of DAUs, we also implemented a 3D-printed case to accommodate the sensing technology together with a 250 mAh Lipo Battery. The setup consists of two Texas Instruments FDC2214 4-channel 28-bit capacitive-digital converters and an Adafruit Feather Sense Microcontroller to gather the data streams and forward them through Bluetooth Low Energy. We further developed a web user interface to connect to the DAU based on JavaScript. The gathered capacitive data from each channel can be collected, visualized, and stored for later usage. Throughout all experiments of this work, we used the same setup of DAU and web user interface for sensing the capacitance. To customize the system for sensing various movements of different body parts, modifications were only applied on the textile level. The connection of the textile to the DAU was established through wired pin connections.

To explore the landscape of the established capacitive sensing principle, we tested multiple combinations of substrates, sensing, and cover materials, as shown in Fig. 1. We built a harness to fix the sample in the textile pocket and thoroughly tested it on the knee and elbow joints. Each conductive part of a sample has a size of 2x10cm in order to obtain comparable results. Most of the samples (a-j) passed our tests of being flexible and sensitive enough to be used for measuring the capacitance change while moving the fabric. However, for sample (b), the low interlocking between the conductor’s mesh resulted in a high resistance and therefore a low conductivity. As a result, samples with resistance values below 10 $\Omega$ are ideal for capacitive sensing whereas higher resistance may influence the measurements. From there on, we also tested different shapes of conductive patches (m-p) which extend the designer’s possibilities for creating a unique sensing garment. Together with the Make-Your-Own-Wearables (MYOW) toolkit, the knowledge from the tested samples can be transferred into smart garments that benefit from the dynamic textile geometry^21,22^. Therefore, the following points form a general guideline for developers and designers on how to build a smart capacitive sensing garment:

- The sensor’s output is correlated to the geometry change of the conductive patches caused by human motion. There are no special requirements on the shapes of the patches, but the designer has to ensure the correct position on the garment to cover the moving area.
- The patches can be placed further away from the small rigid sensing module through conductive traces. This separates the capacitive measurement location from the sensor’s position. Further, the whole trace can be used for sensing to cover larger areas.
- There are minimum technical constraints: a single sensing module can only connect to a fixed maximum number of patches and a single DAU can only connect to a fixed maximum number of modules. So it is best if the patches are routed in bundles to the sensing unit. For the MYOW toolkit each bundle is limited to four patches and each microcontroller can only handle two bundles^21^.

## Joint angle measurement

In order to prove the usefulness of textile capacitive sensor patches’ size, position, shape, and number of channels, initial tests that emphasize the correlation between human body movement and the capacitive signal were necessary. A common practice to evaluate a new sensing technology for its capabilities in this scope is single joint angle measurements^23–25^. To check the precision of our capacitive sensing technology, multiple patterns were designed, and based on that, we created three prototypes. They were then tested to assess which of them returned the best outcomes.

**Figure 4:**
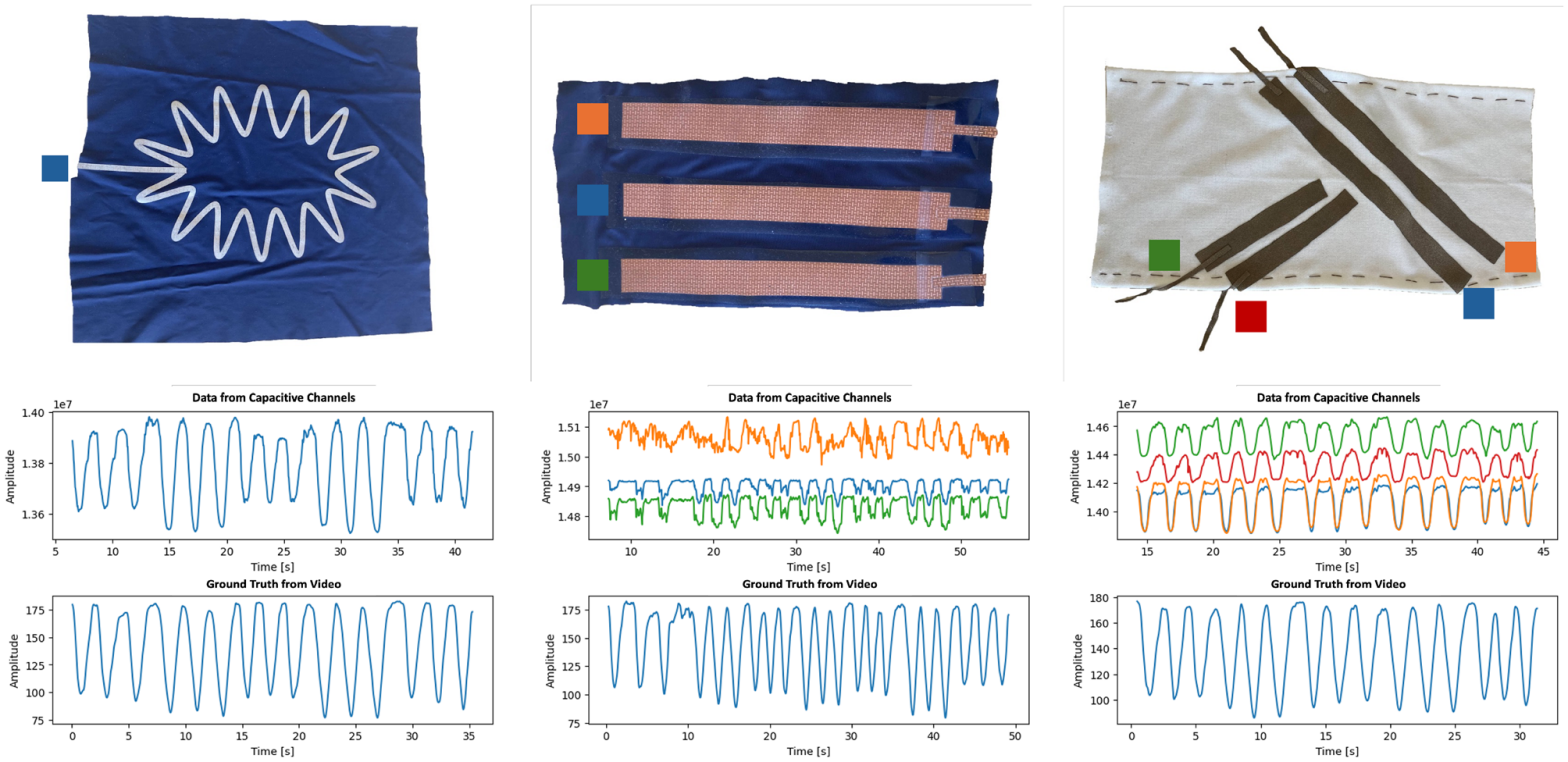
The correlation between the selected capacitive sensor patches with one, three, and four channels and the ground truth from video tracking. Each capacitive channel is assigned with a different color to highlight the signal stream with the placement and shape of the patch.

In Fig. 1k,l, two special harnesses for testing the prototypes are presented. They can be mounted on the knee joint which allows for easy changes between sessions. Both of them are created from two pieces of fabric in between which there is a pocket for the created designs. Red markers on its side were used for generating the ground truth through single-camera video tracking by calculating the angle of the knee movement. Through Fig. 4, three tested patterns are presented. The first prototype consists of one piece of conductive material arranged in the form of a wavy oval. This shape surrounds the center of the joint which has the potential to collect valuable data as it accumulates all of the environment changes in one signal. In the second design, the three stripes are placed evenly in a vertical way. As the harness is placed directly on the top of the knee, this arrangement could gather information about the bending of the knee as it would stretch the conductive material generating a stronger and clearer signal. The last prototype has four diagonally placed stripes. In this way each one of them would be affected by the smallest movement of the leg, be it flexion and extension or lateral rotation. Another difference between all three of the designs is the material they are made of: only the last one is flexible which essentially is more comfortable for users and should be more resistant against mechanical fatigue.

To prove the concept of the potential usability of capacitive sensors for human motion capturing, we conducted an experiment to gather data from the capacitive prototypes and the video tracking setup. The experiment was set up to prove that the data gathered by capacitive sensors resembles the movement of the leg, wherefore two types of motion were chosen: squats and a less dynamic version of ”high knees”. The first one starts in a standing position and then the exercise is performed until the angle of the knee gets to 90 degrees; then the person stands up and finishes 5 seconds after the angle gets to 180 degrees. The second one also starts while standing and then one leg is raised forward with a bent knee until its angle gets to 90 degrees, then the leg should be put down on the ground. Each prototype was set up to the already introduced DAU to forward and store the capacitive data for later synchronization with the ground truth video tracking.

As shown below the utilized patches in Fig. 4, sample sections of each prototype are visualized through the alignment of the capacitive channels next to the video-based extraction of the knee angle as ground truth. We added the color of each capacitive channel from the graph in the patch images. The prototype with one channel only already shows a reasonable signal, whereas the other two prototypes show much finer movements due to their multi-channel layout to introduce more signal robustness. Moreover, the multi-channel prototypes further enable the measurement of joints with more than one degree of freedom by adjusting the layout of each channel to match the corresponding motion. Such collected and synchronized data stream can then be further applied to various machine learning applications to benefit from the strong correlation between the angular motion and the capacitive signals. Especially when utilizing multiple channels with different sizes and alignments, an increased granularity of information can be obtained from each channel to enhance the correlation robustness between capacitance and the ground truth from the video.

## Full body capturing

Previous works have already presented possible ways to transition from linear joint angle measurements toward larger body segment tracking. Instead of measuring one angle only, the interaction of multiple joints can be tracked through larger wearable systems like capacitive sensing jackets for the upper body. Bello et al.^26^ created a blazer equipped with four capacitive patches to detect 20 different arm and torso movements. A classifier, trained on the gathered dataset of fourteen recorded participants can predict the selected gestures with 97.18% accuracy for leave one recording out and 86.25% accuracy for user-independent recognition. Similar work was conducted by Zhou et al.^19^ through a sportswear jacket equipped with eight capacitive patches to sense arbitrary arm and upper body movements. A 38-h dataset of synchronized video and capacitive data from 21 participants has been recorded to train a deep neural network regression model to continuously predict the 3D upper body joint coordinates of the user. The system achieves satisfying results with $R^2$ = 0.91; MPJPE (Mean-Per-Joint-Position-Error) = 86 mm and additional F1 score = 0.9 for 10-class classification with unsupervised class discovery based on the loose-fitting capacitive sensing garment.

In general, it is up to the user to select the arrangements and design of capacitive sensing patches due to the low technical requirements. Multiple strategies are possible, e.g. giving importance to joints with multiple channels for higher accuracy, or less with only one small patch. Moreover, the shape of the patch can be designed to detect the full joint or parts of complex joint movements only for instance shoulder or hip joint movements. The size and shape can also be used to define focal points, for example, to highlight regions with weaker movements. Considering larger tracking areas, the freedom for designers to embed the tracking technology into the garment without restricting the functionality increases. The following sections present ways to merge the technical approaches to capture body movements with a design-oriented approach to incorporate the introduced guidelines for seamless textile integration.

### Laser cut digital art

**Figure 5:**
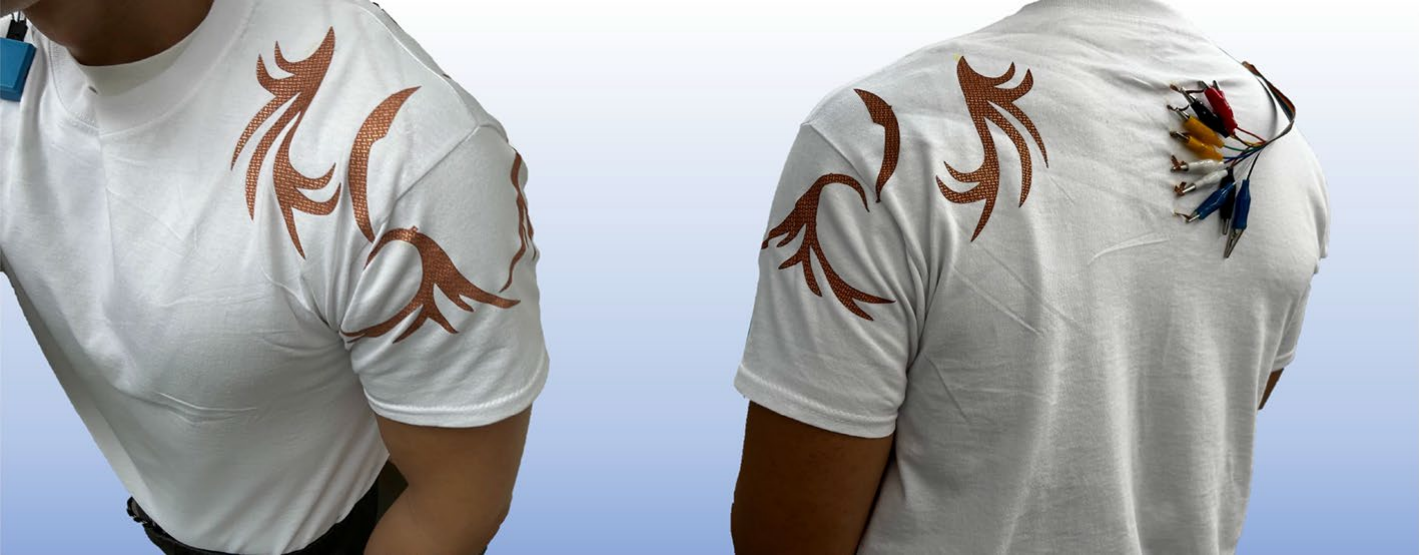
Laser cut vector art from digital artist connected through hidden traces on the garments inside.

We asked a digital artist to create some abstract geometry to transition from technical prototypes as shown before to design-centric smart wearables. With a focus on the shoulder, the design follows the natural movement of the shoulder joint, so that each part of the design can be connected to one separate channel to independently capture the complex shoulder rotation. The designer’s vector art can be converted into capacitive sensing patches using a laser cutter for the conductive fabric and a programmable plotter for the isolation foil. The produced design components are heat-pressed onto a plain shirt afterwards, following the introduced methodology. To express the sensor patches as prominent design elements, they can be installed on the outside of the garment, and the conductive traces, used for the wiring towards the DAU, can be routed on the inside of the garment. From our testing, the placement on the inside or the outside of the garment does not matter since the isolation foil minimizes the influence of touching the skin on the garments inside and helps to sense the deformation only. Figure 5 shows the final implementation of the design concept into a fully integrated smart wearable, being able to sense the wearer’s shoulder movement with capacitive sensing technology.

The semi-automated workflow through programmable cutting and plotting machines promotes the diffusion of the technology as any designing enthusiast can transform their vision into motion-sensing garments. Using vector bitmaps, the designs can also be easily plugged into 3D design software such as Clo3D or Blender to visualize the design in dynamic 3D environments to iteratively fine-tune design aspects (e.g. shapes and placements) before implementation. This approach can also be replicated with processes similar to current online shirt printing services.

### Design-centric full body capturing suit

**Figure 6:**
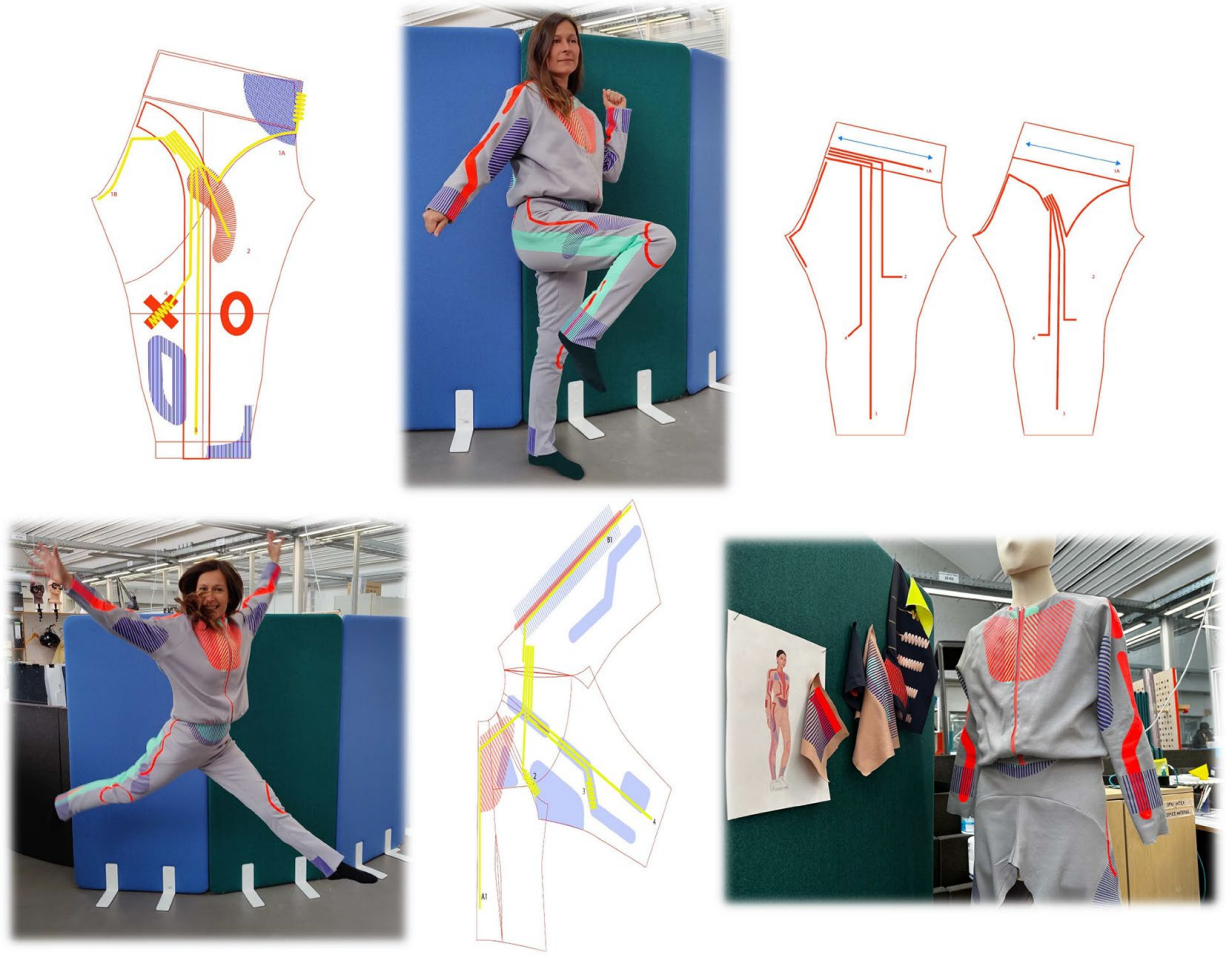
Design process of embedding the capacitive sensing technology into a custom-built suit by a professional experienced fashion designer.

A major barrier for professional fashion designers when they want to integrate technologies into their smart wearables is the uncertainty of whether the design has a negative effect on the technology without the close guidance of technical experts. This uncertainty greatly restricts the creative process. With the capacitive sensing technology, designers have simple rules to follow as explained above which encourages them to focus on the creative process and the dynamic geometry changes of the fabric. We invited a professional smart fashion designer to express her design ideas regarding a full-body motion-capturing suit. While it is difficult to describe the aesthetics and inspiration aspects in words, we describe the implementation process below and show the overall design in Fig. 6. The conductors are integrated into the fabric on the left-hand side, i.e. the inside. This approach has the advantage that the conductors are almost invisible in the garment. They are insulated with thermal transfer film, shielding them from skin contact. The tracks run parallel for most of the distance, which has the advantage that the ends of the tracks act as data input first, as most of the tracks run parallel and generate similar data. Some tracks have an antenna element at the ends that runs in curves. Especially in areas where diffraction causes wrinkles, the area of data collection can be spread over a larger area. We have also developed insulation for these elements and studied which types of curves remain the most elastic. The insulated tracks can be printed with decorative elements on the other side of the textile. We opted for a design that follows the course of the conductors and also laminates the conductors on the right-hand side of the fabric. The patterns follow the positions of the tracks and sensors. However, it is possible to change the pattern to suit custom requirements.

## Hand and finger pose detection

Since it is in the nature of humans to control things by hand with a high level of precision, there is emerging interest in smart wearables to track hand and finger movement, mainly coming from the human-robot interaction (HRI)^27,28^. Due to the hand’s biological structure being very sophisticated because of many joints for executing the essential and versatile movements of the hand, the tracking technology must also meet higher requirements in order to work reliably. In the following, we present two current research projects aiming to use capacitive sensing technology for tracking the hand and fingers through a smart glove. Both prototypes implement the introduced capacitive sensing technology, the first one combining it with an IMU to realize gesture recognition for drone controls, whereas the second glove is designed to reconstruct the wearer’s finger pose through a regression model.

### Gesture recognition

**Figure 7:**
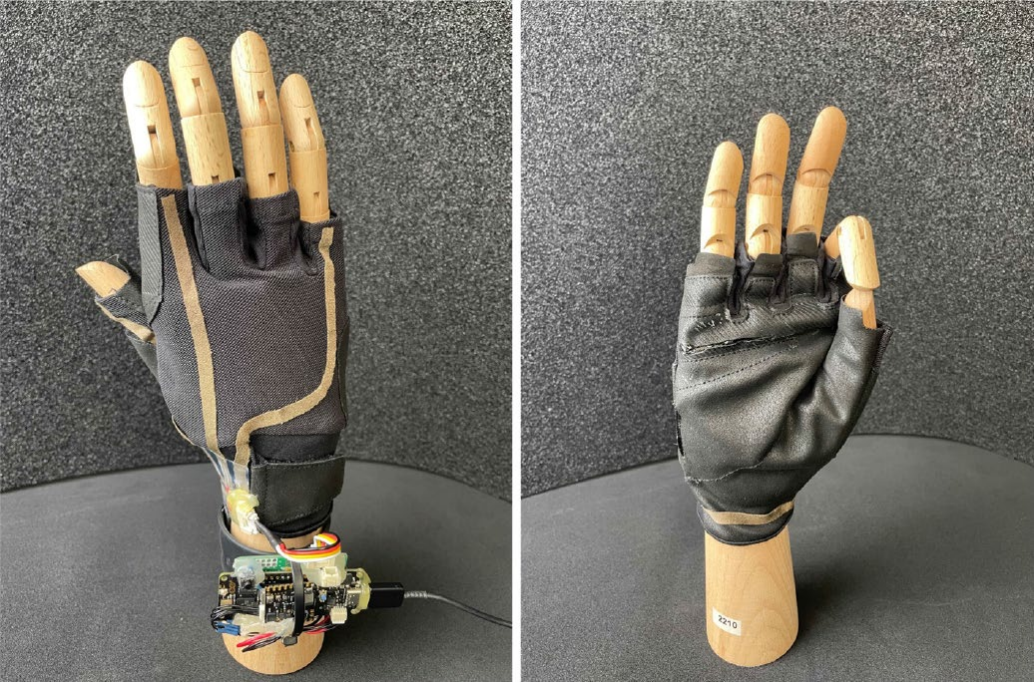
Gesture recognition glove with capacitive sensing and IMU. The wristband hosts the embedded board to realize real-time and on-the-edge (RTE).

The gesture recognition as shown in Fig. 7 glove is built from a sports glove that does not cover the entire fingers to maintain the user’s mobility and comfort. It is equipped with four stretchable and soft capacitive electrodes to sense the finger movements and an IMU placed on the wrist, delivering the three-axis orientation. Together, they implement a capacitive and inertial fusion-based glove-based design for real-time on-the-edge hand gesture recognition. The main board is a Portenta H7; the main processor is the dual-core STM32H747, including a Cortex M7 running at 480 MHz and a Cortex M4 running at 240 MHz. Portenta H7 offers 2MB flash and 8MB SDRAM and wireless data transmission options such as WiFi, Bluetooth classic, and BLE. The inertial board is a Nicla Sense with a 64 MHz ArmCortex M4 (nRF52832) and sensors such as; IMU, air pressure, humidity, temperature, and gas. Nine hand gestures are defined, which can be used for basic drone controls as a downstream application (Up, Down, Back, Forward, Land, Stop, Left, Right, Null). Based on the hierarchical fusion of inertial and capacitive information, the aim is not only to use capacitive sensing as an alternative to state-of-the-art computer vision solutions. Moreover, we try to reduce the power requirements and provide tiny memory models suitable for real-time on-the-edge (RTE) devices to support the development of standalone smart wearables further.

**Figure 8:**
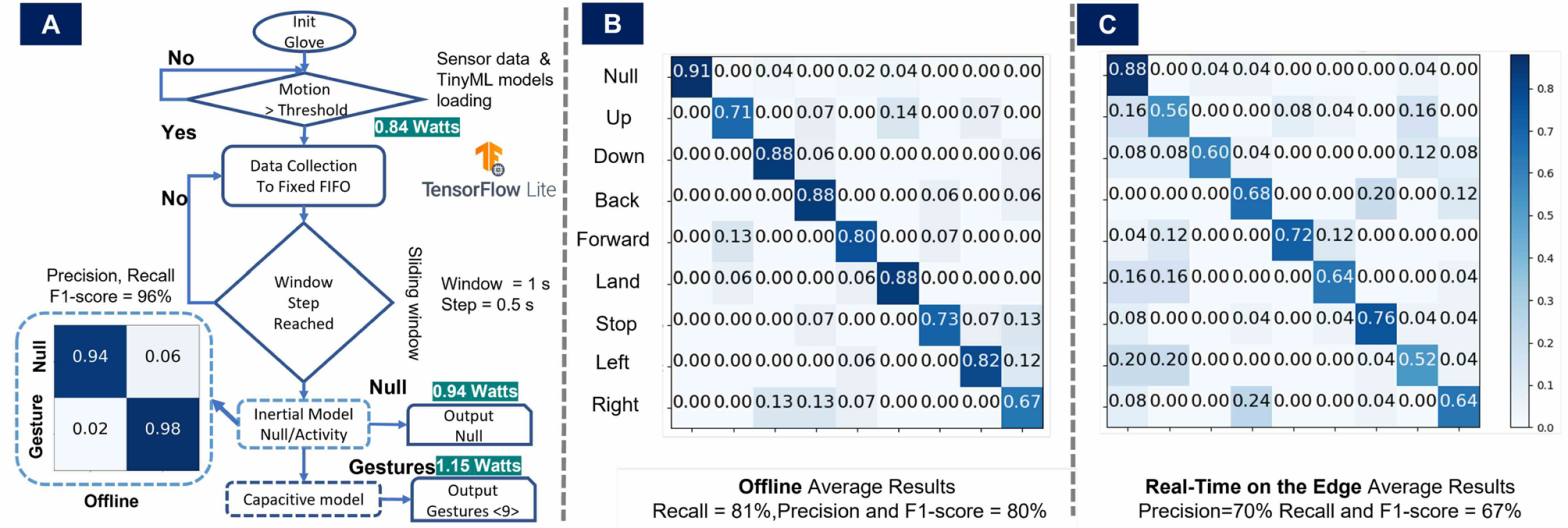
(**A**) Real-time on-the-edge (RTE) Implementation for Hand Gesture Recognition with two stages, first the detection of gestures, second the classification of gestures, both with focus on low energy and memory footprint. (**B**) The average offline results for final classification. (**C**) The average RTE results, deploying the models on the main board Portenta H7 and the inertial board Nicla Sense.

As shown in Fig. 8A, two collaborative models were deployed for gesture recognition. Pre-normalization per window is applied to the inertial and capacitive signals. The window size is 2 seconds, and the window’s step is 0.5 seconds. The first neural network model is the inertial model with three channels as input (linear acceleration). This model obtains a Convolutional Neural Network (CNN) architecture and is used to distinguish the null class from gesture detection. The null class includes activities such as; walking and standing/sitting down, among others. The output of the acceleration model served as a trigger for the second model, the capacitive model. If an activity is classified as non-null, the capacitive model is activated. The second model uses a CNN architecture as well by fusing the four capacitive channels as four independent input channels. The outputs of the capacitive model are the nine classes as listed in Fig. 8B.

For the training data (offline results), one volunteer (female) participated and mimicked (randomly ten sessions) the selected gestures while wearing the system. The sessions were recorded on different days to ensure that our device was not worn repeatedly. The offline evaluation scheme was 10-fold cross-validation with a leaving-one-session-out. Each session had four random tries per gesture.

The F1-Scores for the offline inertial model was 96%, the offline capacitive model resulted in 80%, and the RTE results (5 sessions, re-wearing, one volunteer) gave an F1-score = 67%. Further, the hierarchical fusion of the inertial and capacitive information impacts the power consumption reduction by about 27%. The fusion method also helps reduce model complexity and parameters to obtain lightweight neural networks to be deployed in embedded devices. To sum up, the confusion matrices for offline and online recognition are presented in Fig. 8. In the offline results, we can observe confusion between the gestures, Up and Land (14%) and Forward and Up (13%); both pairs mainly differentiate by how the finger’s upper parts move. The sports glove we employed does not cover the finger’s upper parts to allow flexibility/comfort for the user. There is also confusion for the case of the pairs; Stop and Right (13%), Right and Down (13%), Right and Back(13%), and Left and Right (12%). All these pairs have in common that the fist is closed, and their main difference is how the thumb and the index finger move. For specific applications such as sign language gesture recognition, the glove can be extended to cover the fingers completely to reduce confusion as shown in the following section.

### Finger skeleton reconstruction

**Figure 9:**
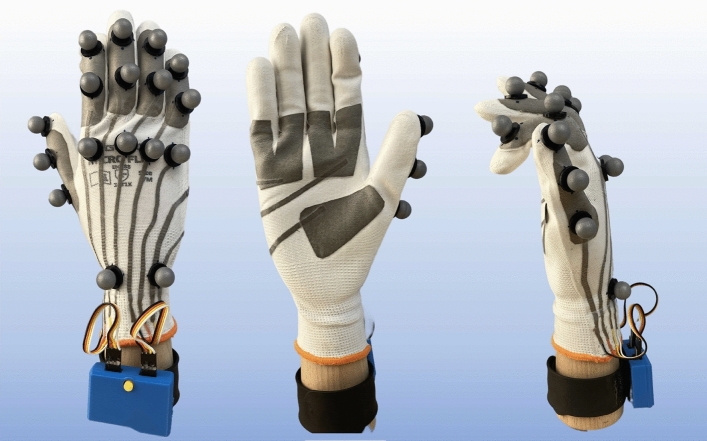
The capacitive glove is equipped with 8 capacitive channels, an additional 17 markers for the ground truth tracking, and the DAU worn on the wrist.

Instead of recognizing predefined gestures for specific use case scenarios, we are aiming for a more generic approach to reconstruct the complete skeletal pose of the hand during arbitrary movements through a capacitive glove. Due to the complex and simultaneously fine movements of the hand, this task forms the spotlight of our work and further stretches the boundaries of capacitive tracking technology.

The implemented glove covers the entire hand and is equipped with eight-channel sensing patches made from flexible conductive fabrics^29^. To maintain flexibility and comfort, the introduced heat pressing technology was applied together with only flexible materials for each layer. As a protection, the isolation layer of the glove is highly flexible and thin, although not very robust to strain and interference, resulting in a trade-off between the wearer’s comfort and signal quality. The routing and placement of these patches can be seen in Fig. 9, the primary objective is to cover the most relevant joints with sufficiently sized patches to obtain distinct signals from the deformation of the conductive patches. We added sensing patches to both the top and bottom of the fingers because, on closer inspection, each finger can perform two basic movements: the patches on the top track the movements of the two smaller finger joints, whereas the patches on the bottom track the movements of the main joint of the fingers.

To produce a dataset suitable for deploying a regression model for continuous hand skeleton reconstruction, an experiment was conducted. The input of the model consists of eight-channel capacitive signals without any additional sensory information. To gather ground truth data, a HoloLens 2 with visual tracking and a Leap Motion infrared sensor placed on top of the lens was set up. Both systems captured the same field of view, resulting in two separate ground truth datasets of the recorded hand. The Mixed Reality Toolkit 2 (MRTK) from Microsoft was utilized to extract the global coordinates for each joint and fingertip with the origin at the Holo Lens’ front^30^. As it turned out after the first test recordings, both generated data sets were not able to extract the hand pose with sufficient precision. A subsequent hand pose estimation from a single RGB image algorithm applied to the video material could not solve this problem either^31^. The issue was not the setup or conditions of the experiment, rather it was the glove itself. Since the video-based tracking systems were trained on the bare hand, the glove leads to a lack of relevant information such as shadows cast or wrinkling of the skin.

To overcome the drawbacks of the previous tracking approaches, an infrared-based Vicon Nexus motion tracking setup, provided by Vicon Motion Systems Ltd. was utilized. The configuration consists of 8 Vero v2.2 cameras, strategically positioned to optimize tracking of the complete range of the hand movements. Reflective markers with a 14 mm diameter, mounted on a 1 mm thick base, were placed on key joints of the hand as outlined in Cook et al.^32^. Operating with a 100 Hz sampling rate, the Vicon system provides a robust and reliable ground truth environment for precise motion tracking, which enables a detailed validation of the capacitive glove’s performance. For each reflective marker, the global 3D coordinates are retrieved from the motion tracking system to further extract the required ground truth information.

The experiment involved recording data from five participants with different hand sizes, ranging from loose fitting to tight fitting, to obtain an unbiased dataset. Each participant performed three sessions, each lasting around 7 minutes, while performing arbitrary finger movements of individual fingers and combinations of them. The instructor followed the sessions and suggested additional movements and gestures based on the participant’s ingenuity to ensure the coverage of the majority of possible movements for building an unbiased dataset. The participants performed the experiment in a sitting position, holding their hands in an upright posture. The movement of the wrist cannot be tracked with the glove and therefore played an irrelevant role in this experiment. Forming a fist three times at the beginning and end of each session served as a synchronization point to align the dataset streams afterward. The resulting dataset spans approximately 100 minutes, with a sampling rate of 30 Hz for the capacitive data of the glove. The marker-based motion tracking system’s recording with 100 Hz was time-synchronized and down-sampled to match the capacitive data.

We recognized a correlation between the capacitive signal and the curvature angle of the fingers during the synchronization of the dataset, where we decided to simplify the skeleton model to one value per finger. The calculation is based on the global coordinates of the marker-based tracking, moreover, we implemented the angle based on the two vectors that are spanned between the three markers located on the finger joints. The greater mobility of the thumb resulted in insufficient angle calculations due to the narrow placement of the markers. Nevertheless, a more promising calculation could be realized by utilizing the wrist marker as the origin to span the two vectors. With this strategy, we are able to incorporate the movement of the thumb metacarpal bone, which typically has larger mobility than the ones of the four fingers.

Compared to using any distance information for the ground truth, the angle is independent of the participant’s hand size as well as from the offset between the joint and the marker’s center point. The final dataset consists of eight-channel capacitive data aligned with the simplified hand skeleton model of five angles.

To evaluate our approach, we developed a regression model based on the common Convolutional Neural Network (CNN) architecture^33,34^. Our architecture consists of three convolutional layers followed by two fully connected layers. Together with dropout layers of 0.5 after each activation, this slim architecture is sufficient to generalize the dataset. Due to some naturally occurring outliers in our capacitive data, we trained the model with the Huber loss function which combines the L1 loss and the L2 loss which results in a model that is less sensitive to outliers while still providing smooth gradients for optimization^35^. By adding a sliding window of 30 data points for the capacitive data, which corresponds to about one second of the recording, we were able to stabilize the model training further.

The model takes five values as input, which is the calculated angle for each finger. Even though the finger representation is simplified that much, we can still train the model well on our dataset due to the correlation between the five data points. From a biological perspective, there are certain restrictions on finger movements due to the anatomy of the hand skeleton. Each finger’s individual movement may seem indefinite, but there are a lot of finger movement combinations where fingers restrict each other. Our model can benefit from that by learning all five fingers at once and incorporating the dependencies, e.g. ring and pinky finger.

**Figure 10:**
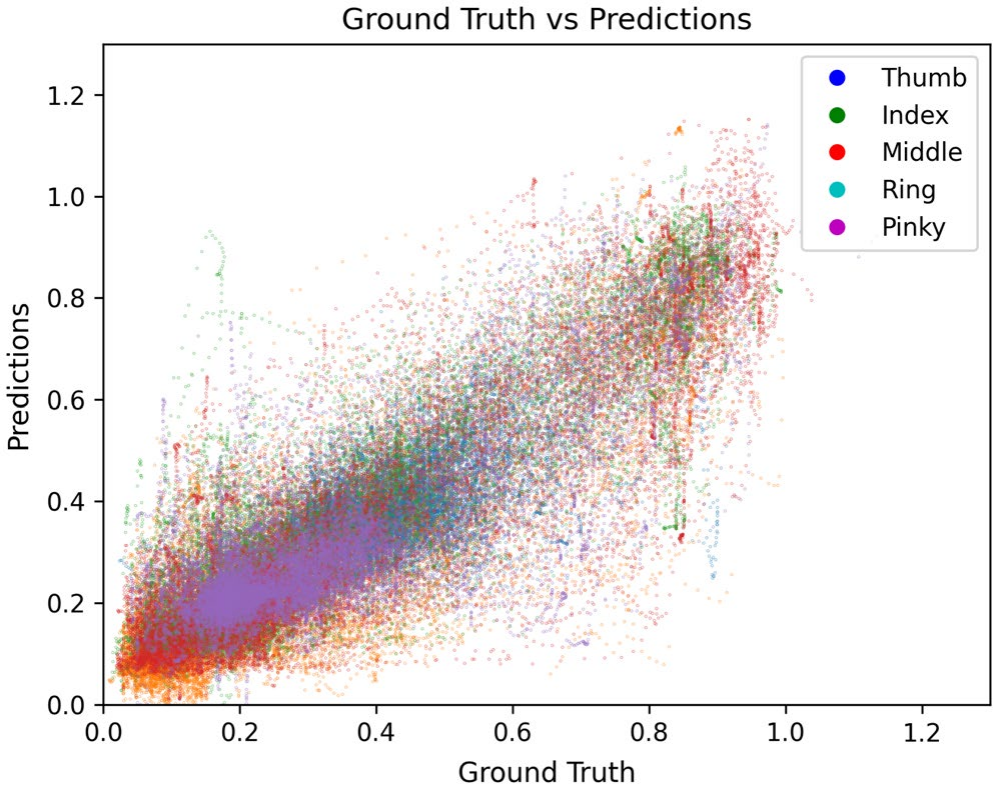
Regression plot of Ground Truth versus prediction across the five fingers, highlighting the range of movement and the accuracy of the prediction for each finger.

We evaluated our model using cross-validation, employing two distinct approaches. In the first approach, the model was trained across all participants, with one of the three sessions each left out for the test and validation. In the second approach, we trained individual models for each participant, leaving out again one of the three sessions. We plotted a regression plot in Fig. 10, visualizing the correlation between ground truth and prediction for each finger in a different color. The majority of predictions align with the ground truth, generating a vertical line in the graph. However, outliers can be found for all fingers. More interestingly, we can also see the range of motion for each finger and the majority of finger positions. For instance, the pinky has the least range of motion. The large cluster in the lower values range shows that most participants had their fingers angled, whereas there is another smaller cluster in the high-value range for the full extended fingers. In between, there are fewer data points present since these values are usually only reached when moving from angled to full extension and back. The first approach resulted in an $R^2$ value of 0.74 and a Root-Mean-Squared-Error (RMSE) of 11 degrees for the average absolute error whereas the second approach resulted in an average $R^2$ value of 0.76 and an average RMSE of 10 degrees across the models trained on the five participants separately.

**Figure 11:**
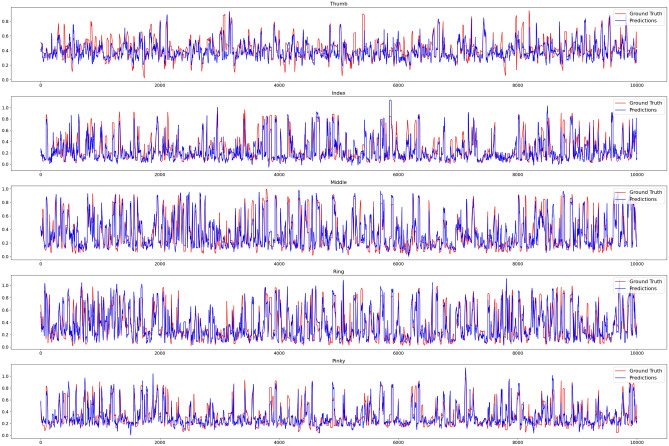
The ground truth representation of the hand (distance between finger knuckle and finger tip) extracted from the marker-based tracking overlaid with the prediction of the trained CNN model for all of the five fingers.

An additional in-depth analysis of each finger showed that the thumb obtains weak results of an average $R^2$ around 0.6 whereas the four fingers ranged between 0.78 to 0.87. This result can be explained by the already mentioned higher mobility of the thumb and the issue of tracking it properly. When comparing the direct correlation for each finger in Fig. 11, there is an enhanced deviation for the thumb, emphasized through the higher occurrence of red lines. Compared to the other fingers, at least following the trend of the ground truth, the model struggles to properly predict the thumb. Moreover, the per-person model training demonstrated that tighter fitting of the glove resulted in a worsened model prediction of up to 3 degrees in the RMSE results, which we can attribute to restricted movement of the garment and accompanying weaker signal.

The results manifest our idea of a loose-fitting garment decoupling the fixed calibration between tracked joint and sensory with additional comfort considerations. Compared to other related works^36,37^ using sensory in as glove to reconstruct the hand skeleton, our significantly simpler design with only 8 capacitive sensing patches produces satisfying results to reconstruct the hand pose based on our five-angle approach.

## Conclusion

In conclusion, we demonstrate multi-channel capacitive sensing as a valid alternative for human motion capturing. Our approach of embedding conductive textile patches into everyday life garments as a foundation for sensing body movements highlights the idea of combining technology with design considerations. The wide variety of materials, shapes, positioning, and the resulting, diverse solutions to motion tracking incorporating design considerations offer many more possibilities than the applications shown here. Textile capacitive sensing can find its application in downstream tasks from various fields, for instance, the medial area for rehabilitation purposes, the sign language community for gesture-to-speech translation, or the film industry to track actors’ movements without visual interference through markers or skin-tight sensors. Moreover, capacitive sensing can be applied in all areas where human body tracking technology has to be operated without personalization or calibration to the user’s body while at the same time maintaining the expected level of comfort for loose-fitting smart wearables.

## Methods

### Sensing principle and interpretation

**Figure 12:**
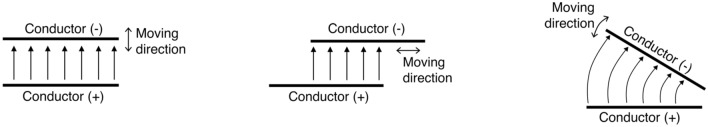
Double-ended (differential) capacitor sensing. The displacement of a pair of capacitor plates influences the effective capacitance.

**Figure 13:**
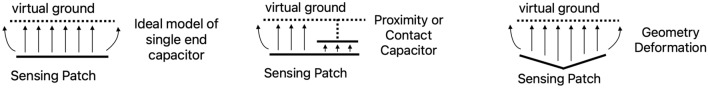
Single-ended capacitor sensing principle with single-end capacitor and virtual ground.

The sensing capacitor can be configured as a double plate capacitor^14^ as shown in Fig. 12. Apart from classic double plate capacitors, we can use only one conductor plate to form a capacitor with the virtual ground in the environment, as a single-ended capacitor^38^. The music instrument Theremin uses this principle to detect the range of the performer’s hand which is considered the virtual ground. The ideal model of a single-end capacitor is shown in Fig. 13 next to the two application scenarios. For the wearable application, the first is proximity or contact to the sensing patch whereas the second is the sensing patch’s geometry deformation. Due to the virtual ground, the technology can be embedded into textiles through a single conductive sensing patch. Single-ended capacitive sensor also has better sensitivity^39^.

**Figure 14:**
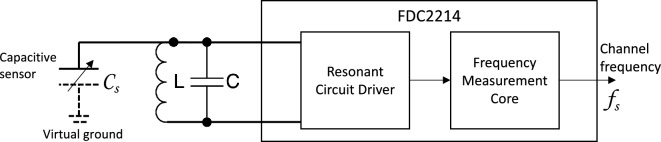
Measuring circuit with FDC2214.

To convert the dynamic changes of the single-ended capacitive sensors, we used the Texas Instrument FDC2214 capacitive-digital converter. The simplified circuit diagram of a single channel is shown in Fig. 14. FDC2214 integrates a resonant circuit driver for each circuit, which excites an LC resonant circuit with an external fixed inductor (*L*) and capacitor (*C*) as passive components on the printed circuit board. The sensing capacitive plate forms a variable capacitor with the virtual ground ($C_s$) connected in parallel with the fixed capacitor, which changes the resonant frequency ($f_s$) of the LC circuit. The resonant frequency of the LC circuit, measured by the FDC2214 core, can be expressed by the equation below:

$$\begin{aligned} f_s=\frac{1}{ 2\pi \sqrt{L(C+C_s)}} \end{aligned}$$

The frequency-based sensing is robust against external or cross-channel electrical interference, as the measurement core counts the frequency of the resonant signal with a peak voltage of VDD (3.3V average). In our application, although 18 uH inductors and 33 pF capacitors were used for all channels; each component has a factory tolerance of 10%, which means the exact values are slightly different and such difference is sufficient to introduce a significant offset for the operating frequencies ($f_s$ when $C_s=0$). Cross-channel interference was not observed.

Deep convolutional neural networks are used to interpret the multi-channel frequency data which represents the dynamic changes of $C_s$. Since the introduced DAU gathers multichannel frequency on different levels, normalization of the recorded data is a crucial preprocessing step. By taking the mean and the standard deviation of each channel separately, each channel can be normalized into the range between 0 and 1. Having all channels on the same range now, the data is independent of the biased operating frequency and can be utilized for synchronization with the ground truth data followed by the training of the model. Since we preprocess each channel independently, also the adaption to the connected textile patch is maintained. We used textile sensing materials from ShieldEx^29^, which maintain their structural properties within their specifications. During our tests, we could not determine any fatigue or changes in material. The employed layer stacking conductive textiles and protective insulation foil ensures the long-term stability of the system. Nevertheless, to integrate potential differences in frequency changes over time, long-term inference with capacitive sensing involves an online normalization process that automatically integrates the degradation of material over time through the mean and standard deviation calculation.

### Ethical agreement

All participants signed an informed consent following the Declaration of Helsinki. The ethical committee of Kaiserslautern University and the German Research have approved the study. Participation was entirely voluntary and could be withdrawn at any time. The participants did not receive any compensation for their participation. The subjects could deny answering questions if they feel uncomfortable in any way. There are no risks associated with this user study. Discomforts or inconveniences will be minor and are not likely to happen. All data provided in this user study will be treated confidentially, will be saved encrypted, and cannot be viewed by anyone outside this research project unless separate permission is signed to allow it. The data in this study will be subject to the General Data Protection Regulation (GDPR) of the European Union (EU) and treated in compliance with the GPDR.

### Image publication agreement

Informed consent was obtained from the involved individuals to use their facial image for this publication, with an understanding that it may be disseminated in print, digital, or online formats for the specified purpose.

## Data Availability

The datasets generated and/or analyzed during the current study are not publicly available due to the confidentiality and data protection of the participants but are available from the corresponding author on reasonable request.
